# Supplementary material for: Identification of photoperiod-induced specific miRNAs in the adrenal glands of Sunite sheep (Ovis aries)
Source: Front Vet Sci. 2022 Jul 22;9:888207. doi: 10.3389/fvets.2022.888207 (PMC9354845; doi:10.3389/fvets.2022.888207)
Supplement: Supplementary file 1 [file Data_Sheet_1.ZIP › Supplementary file/miRDeep2 file.zip!/miRDeep2 file/Know_Structure/SP42A3/oar-mir-194.pdf]

miRBase precursor	: oar-mir-194
Total read count	: 3316
oar-miR-194 read count	: 3308
remaining reads	: 8

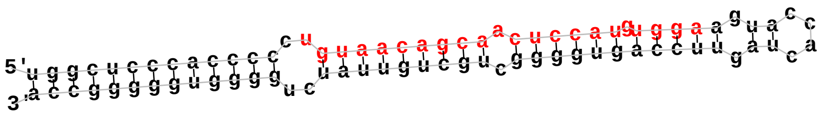

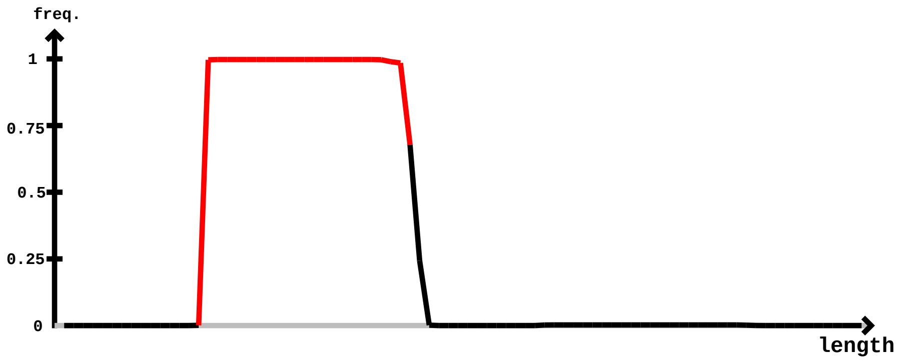

oar-miR-194

```
5'- uggcucccacccccuguaacagcaacuccaugugga aguaccacuaguuccaguggggcugcuguuaucuggggugggggcca   -3'    exp
    (((((((((((((..(((((((((.((((((.(((((.((....)).))))))))))).)))))))))..)))))))))))))   reads      mm         sample
    .............cuguaacagcaacuccaugug.................................................   1          0          seq
    .............cuguaacagcaacuccaugugga...............................................   2          0          seq
    ..............uguaacagcaacuccaug...................................................   3          0          seq
    ..............uguaacagcaacuccaugu..................................................   24         0          seq
    ..............uguaacagcaacuccaugug.................................................   15         0          seq
    ..............uguaacagcaacuccaugugg................................................   1017       0          seq
    ..............uguaacagcaacuccaugugga...............................................   1436       0          seq
    ..............uguaacagcaacuccaugugga C..............................................   17         1          seq
    ..............uguaacagcaacuccauguggaa..............................................   784        0          seq
    ..............uguaacagcaacuccauguggaag.............................................   5          0          seq
    ...............guaacagcaacuccaugugg................................................   1          0          seq
    ...............guaacagcaacuccaugugga...............................................   3          0          seq
    .................................................ccaguggggcugcuguuaucu.............   3          0          seq
    .................................................ccaguggggcugcuguuaucug............   3          0          seq
    .................................................ccaguggggcugcuguuaucugg...........   1          0          seq
    ..................................................caguggggcugcuguuaucug............   1          0          seq
```
